# Immunogenomic-Based Analysis of Hierarchical Clustering of Diffuse Large Cell Lymphoma

**DOI:** 10.1155/2022/9544827

**Published:** 2022-08-09

**Authors:** Longhao Wang, Wei Yuan, Lifeng Li, Zhibo Shen, Qishun Geng, Yuanyuan Zheng, Jie Zhao

**Affiliations:** ^1^Internet Medical and System Applications of National Engineering Laboratory, The First Affiliated Hospital of Zhengzhou University, China; ^2^Department of Pharmacy, The First Affiliated Hospital of Zhengzhou University, China; ^3^People's Hospital of Henan University of Chinese Medicine, China; ^4^People's Hospital of Zhengzhou, Zhengzhou, 450052 Henan, China

## Abstract

Diffuse large B cell lymphoma (DLBCL) is one of the most usual types of adult lymphoma with heterogeneousness in histological morphology, prognosis, and clinical indications. Prior to this, several studies were carried out to determine the DLBCL subtype based on the analysis of the genome profile. However, classification based on assessment of genes related to the immune system has limited clinical significance for DLBCL. We systematically explored the DLBCL gene expression dataset and provided publicly available clinical information on patients with GEO. In this research, 928 DLBCL samples were applied, and we calculated 29 immune-related genomes' enrichment levels in each sample and stratified them into high immunity (Immunity_H, *n* = 135, 28.7%), moderate immunity (Immunity_M, *n* = 135, 28.7%), and low immunity (Immunity_L, *n* = 12, 2.6%) that was based on ssGSEA score. The ESTIMATE algorithm was used to calculate stromal scores (range 586.88 to 1982.43), immune scores, estimated scores (range 2,618.2 to 8,098.14), and tumor purity (range 0.216 to 0.976). All of them were significantly correlated with immune subtypes (Kruskal-Wallis test, *p* < 0.001). At the same time, the correlation of related genes was analyzed by immunohistochemistry staining. In addition, DLBCL cells were cultured in transfected and in vitro with siRNA to verify correlation analysis and gene expression. Finally, human peripheral blood lymphocytes were incubated with DLBCL cells and stained. Flow cytometry was applied to analyze genes' influence on immune function. By analysis, immune checkpoint and HLA gene expression levels were higher in the Immunity_H group (Kruskal-Wallis test, *p* < 0.05). The levels of Tfhs (follicular helper T cells), monocytes, CD8^+^ T cells, M1 macrophages, M2 macrophages, and CD4^+^ memory-activated T cells were the most excellent in Immunity_H, and the total survival rate was higher in the Immunity_L. Through analysis, IRF4 (MUM1) was identified by us as immunotherapeutic target and a potential prognostic marker for DLBCL, which was made sure by using molecular biology experimentations. To conclude, immunosignature made a connection between DLBCL subtypes playing a position in DLBCL prognostic stratification. Immunocharacteristics-related DLBCL subtypes' construction predicts expected patient results and supplies conceivable immunotherapy candida.

## 1. Introduction

Diffuse large B cell lymphoma (DLBCL) is the most common subtype of non-Hodgkin lymphoma (NHL) in the United States, accounting for about a quarter of NHL cases [1]. Two molecularly different DLBCL's shapes have been identified through gene expression patterns including germinal center B cell-like (GCB) types and activated B cell-like (ABC) [2]. The immunohistochemical (IHC) expression of CD10, IRF4/MUM1, and Bcl-6 have been used to categorize DLBCL's examples into non-GCB groups and GCB. Relevant researches have revealed that IRF4's overexpression is connected with patients' unfavorable prognosis with DLBCL. In spite of the variety of clinical, morphologic, and molecular human malignancies used to be classified by parameters nowadays, DLBCL patients' 40% survival continues to be poor.

Up until the present moment, there are few treatment alternatives for DLBCL. Immunotherapy is a new treatment that improves the survival prospects of DLBCL patients, including the blocking of immune checkpoints [3]. In spite of the fantastic advance in immunotherapy strategies, favorable effects, nevertheless, have been demonstrated merely in a subset of patients. Immunotherapy's responsiveness is influenced by definite factors, for example, host germline genetics, PD-L1 grades, and tumor genomics [4, 5]. It has been discovered that tumor microenvironmental heterogeneousness can be used as biomarkers for prognosis and immunotherapy sensitivity of various kinds of cancers [6, 7]. It is noteworthy that both tumor-associated stromal cells and infiltrating immune cells are significant components of tumor immune microenvironment and drama a significant part in tumor development, progression, and drug opposition [8, 9]. In consequence, an increasing number of researches are concentrating on these factors, supplying fresh perceptions into the prognostic value and therapeutic methods of tumor biology.

In our research, based on immune genomic analysis, patients were divided with DLBCL into three groups: Immunity_L, Immunity_M, and Immunity_H. A strong connection has been demonstrated by us between categorization and immune infiltration and survival results. The construction of immune signatures that are associated with DLBCL subtypes may contribute to the search for prognostic markers and novel immunotherapy marks.

## 2. Materials and Methods

### 2.1. Data Source

DLBCL patient of gene expression and clinical data were downloaded from Gene Expression Omnibus (GEO) database (GSE117556). In this research, clinical data that were related to age, stage, subtype, LDH, IPI, ECOG, and survival were collected by us from GEO, and a total of 928 DLBCL patients were enrolled.

### 2.2. Hierarchical Cluster Analysis of DLBCL

29 immune-related gene sets which were widely used in previous studies were applied by us, including 707 genes, depicting different immune cell types, pathways, and functions (Supplementary Table (available here)) [10, 11]. The enrichment grades of 29 immune-related gene sets were worked out by using single-sample gene set enrichment analysis (ssGSEA), as demonstrated in former learnings [10], and quantified by immune cell types, pathways, and functions. DLBCL was hierarchically clustered by using unsupervised machine learning approach and further divided into high immunity (Immunity_H), moderate immunity (Immunity_M), and low immunity (Immunity_L) that be based on ssGSEA score.

### 2.3. Calculation of the Immune and Stromal Scores and Estimation of the CIBERSORT

ESTIMATE is an approach to infer tumor purity's fraction by using immune cells and stromal cells in malignancy tissue applying expression data. In the light of the Immunity_H, Immunity_M, and Immunity_L groups, ESTIMATE algorithm was applied to estimate the immune grade, stromal grade, and tumor purity of DLBCL patients. CIBERSORT is a biological method of Cell-type Identification By Estimating Relative Subsets of RNA Transcripts (https://cibersortx. stanford.edu/). The CIBERSORT package was applied to calculate immune cell types' distribution in each subset, and immune cell's proportion types in DLBCL's subtypes was compared based on the Kruskal-Wallis test, and “∗∗∗,” “∗∗,” “∗,” and “ns” indicate *p* < 0.001, *p* < 0.01, *p* < 0.05, and *p* < 1, respectively [12]. ESTIMATE and CIBERSORT package in R version 3.6.2 (https://www.R-project.org/) are used in this article.

### 2.4. GO and KEGG Pathway Enrichment Analysis

GSEA package was used to analyze gene aggregation and enrichment in DLBCL patients. Gene Ontology (GO) and the Kyoto Encyclopedia of Genes Genomes (KEGG) analyses were used to evaluate the differentially expressed genes' functional function between the low and high group [13]. Differential gene set enrichment was examined using the limma R package. *p* < 0.05 was used as the cut-off value.

### 2.5. Survival Analyses

The Kaplan–Meier survival curve was drawn based on the patient's survival information to visualize the survival difference between immune subtypes.

### 2.6. Cell Lines and Cell Culture

Human DLBCL cell line (DS) is a gift from Professor Mingzhi Zhang, Oncology Department, The First Affiliated Hospital of Zhengzhou University (purchased from ATCC through Genetimes ExCell Technology, Inc. Shanghai, China, ATCC Number: CRL-3381), and used after cryopreservation and thawing. They were cultured in RMPI 1640 medium (Gibco, USA) supplemented with 10% fetal bovine serum (FBS, Gibco, USA), streptomycin, and penicillin in 5% CO_2_ humidified chamber, at 37°C.

### 2.7. Using siRNA to Interfere with Gene Expression

DS cells were transfected with synthetic siRNA oligonucleotides (concentration:100 nmol/L). Lipofectamine 8000 (Beyotime, China) was used by GenePharma (GenePharma, Shanghai, China) [14]. The sequence of siRNA is as follows: siIRF4: 5′-GGACACACCUAUGAUGUUAUU-3′; and siControl, 5′-UAAGGCUAUGAAGAGAUACUU-3′ [15].

### 2.8. RNA Extraction and Quantitative Real-Time PCR

DLBCL cells were inoculated into transfected and cell culture dishes with siRNA oligonucleotide. After transfection with siIRF4 or control siRNA for 48 h, the PrimeScript™ RT Kit with gDNA Eraser (CAT. No. RR047A, Takara, Dalian, China) was reversed transcription following instructions of manufacturer. After transcription, cDNA was quantitatively analyzed applying QuantiNova™ SYBR Green PCR kit (Cat. No. 208054, QIAGEN, Germany) and real-time PCR system (Applied Biosystems, Foster City, California), pursuant to instructions of the producer. For each sample, the mRNA abundance was normalized to the quantity of GAPDH. Primers are as follows: IRF4: Forward, 5 ′-CTACACCATGACAACGCCTTACC -3′ and reverse, 5-GGCTGATCCGGGACGTAGT -3′. GAPDH: Forward, 3′ - AAAGGGTCATCATCTCTG -5′, reverse,5 ′- GCTGTTGTCATACTTCTC -3′.

### 2.9. Western Blotting Assay

DLBCL was inoculated into cell culture dishes and transfected with siControl and siIRF4 oligonucleotides, respectively. After 48 h, cells were collected and lysed with cell lysate for western blotting analysis. BCA assay was used to determine protein concentration, and primary antibody was used: anti-IRF4 (Cat. No. 62834) and anti-PD-L1 (Cat. No.13684) (Cell Signaling Technology, Inc., Boston, MA). The primary antibody was diluted at 1 : 1000 and incubated overnight (12-18 h) at 4°C. And wash with TBST (Tris-Buffered Saline-Tween 20) 4 times, 5 minutes each time. Incubate with peroxidase-labeled 1 : 5000 or secondary peroxidase-labeled goat antimouse IgG (diluted ZGSB Bio, Inc., China) for 2 hours at room temperature. ECL kit (Beyotime, China) was applied for membrane detection. All proteins were loaded with GAPDH as control.

### 2.10. Flow Cytometry Analysis

We directly incubated cells with fluorescent-labeled antibodies and performed cell fluorescence analysis using flow cytometry (BD FACS Canto) to ascertain cell phenotypes for assessment. CD8 and PD-L1 can be stained straight on the cell surface and discovered, while granular enzyme B and IFN-*γ* are employed for intracellular staining, so cells are foremost immobilized with 4% paraformaldehyde for 20-30 min, then stained with fluorescent-pigment-labeled antibodies, and incubated in dark ice for 15 min. Antibody choice FITC-conjugated anti-Granzyme B antibodies and anti-IFN-*γ* were stained with PE-conjugated anti-PD-L1, PE-Cy7-conjugated anti-CD8, and APC-conjugated cells (BD, USA).

### 2.11. Immunohistochemistry (IHC) Staining of Human Diffuse Large B Lymphoma Tissue Array

We purchased human diffuse large B lymphoma tissue Chip array (OD-CT-LY02-001) from Shanghai Outdo Biotech Co. Ltd. Od-CT-LY02-001 Chip array was purchased from Shanghai Outdo Biotech Co., Ltd. It included 30 cases from different parts of the digestive tract and different DLBCL (stomach, small intestine, cecum, colon, etc.). The webpage link of this array is (https://www.superchip.com.cn/biology/tissue.html). IRF4 antibody (Cat. No. 62834, Cell Signaling Technology) and PD-L1 antibody (Cat. No. 13684, Cell Signaling Technology) were stained by IHC to detect the expression of IRF4 and PD-L1 (1 : 50 dilution) [16]. Briefly, 4 *μ*m of tissue array sections were blocked with dehydrated peroxidase. Antigen recuperation was executed at 0.01 mol/L in citrate buffer and autoclaved. The primary antibody was added and incubated overnight at 4°C. Following washes with phosphate-buffered saline (PBS) and incubation with a labeled polymer-HRP second antibody for 30 min, 3, 3-diaminobenzidine tetrachloride (DAB) was applied to initiate the colorimetric reaction. Slides were restained with hematoxylin. The stained slides were observed by microscopy to obtain images. IHC scoring was also performed separately to analyze the correlation between IRF4 and PD-L1. IHC kit was purchased from Absin (Cat. No. abs957).

### 2.12. Statistical Analysis

The enrichment analysis was conducted applying R (3.5.2), and Kaplan-Meier plots were generated using the R package of the survey. ESTIMATE algorithm and CIBERSORT package were applied in R, and Kruskal-Wallis test were applied to compare 22 immune cell types in DLBCL's subtypes. Based on the ANOVA, “∗∗∗,” “∗∗,” “∗,” and “ns,” respectively, indicate *p* < 0.001, *p* < 0.01, *p* < 0.05, and *p* < 1, to demonstrate the HLA family genes' expression in the Immunity_H, Immunity_M, and Immunity_L groups. The GraphPad Prism 8 software (GraphPad Software, Inc., La Jolla, CA, USA) was used to evaluate the statistical significance between two groups. The comparison of parameters between groups was analyzed using *t*-test. “∗,” “∗∗,” and “∗∗∗” represent that the difference between the two experimental groups was statistically significant (*p* < 0.05, *p* < 0.01, and *p* < 0.001).

## 3. Results

### 3.1. Construction Is Modeled by Immune Subtype and Patient Clinical Characteristics

**Figure 1:**
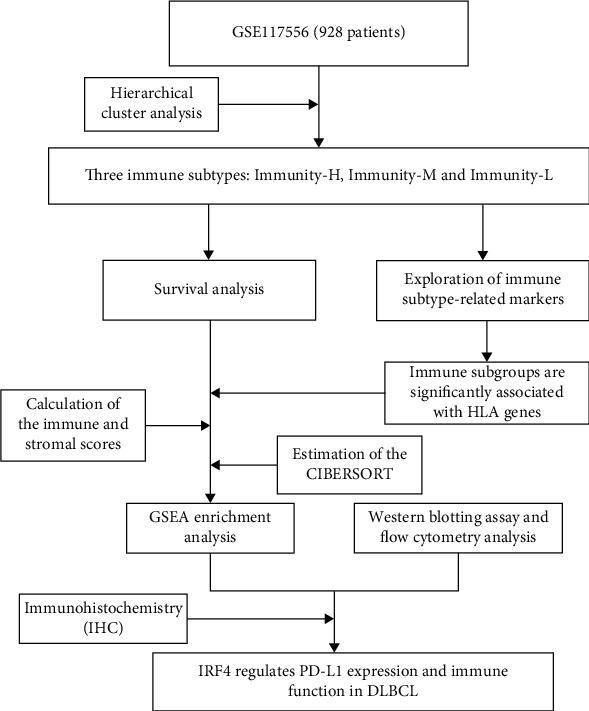
The follow diagram of this study.

**Figure 2:**
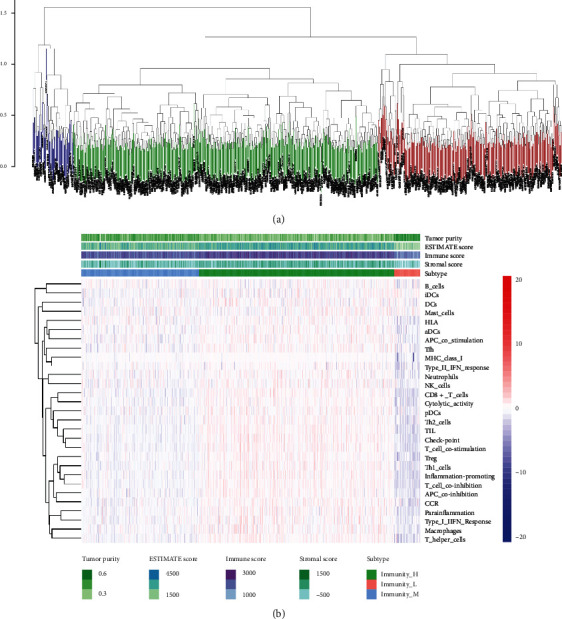
(a) Based on unsupervised cluster analysis of genomic ssGSEA score, 928 DLBCL samples were divided into three groups: Immunity_H (*n* = 636), Immunity_M (*n* = 322), and Immunity_L (*n* = 71). (b) Heat map of Immunity_H, Immunity_M, and Immunity_L subtypes according to 29 immune cell types.

In this study, we involved clinical data and gene expression profiles of 928 patients with DLBCL from the GEO database. The selected patients' clinical characteristics are summarized by Table 1. 62.2 years was the median age at diagnosis (range: 20.8–86.0), with 517 males (55.7%) and 411 females (44.3%). We conducted the study according to the scheme flow in Figure 1. An unsupervised cluster analysis of 29 immune-associated gene sets was foremost performed by us. There were three clear sets of samples according to the ssGSEA score of the genome: Immunity_L (*n* = 71, 7.7%), Immunity_M (*n* = 322, 34.7%), and Immunity_H (*n* = 535, 57.7%) (Figure 2(a)). As demonstrated in the heat map (Figure 2(a)), immunity-related genes' expression degree was more depressed in the low group than in the high group. Stromal scores (range 586.88 to 1982.43), immune scores (range 832.23 to 3359.60), estimate scores (range 1387.54 to 4737.90), and tumor purity (scope 0.27 to 0.69) are revealed for patients with DLBCL. Immune scores and stromal scores were worked out to forecast the level of infiltrating immune cells and mesenchymal and to provide a basis for inferring tumor purity in the tumor tissue (Figure 2(b)). Results demonstrated that tumor purity was importantly more down in the Immunity_H group and substantially more excellent in the Immunity_L group (Kruskal-Wallis test, *p* < 0.001), indicating that this immunotyping correlation analysis with tumor purity in DLBCL is meaningful.

### 3.2. Survival Rate Was Significantly Correlated with Immune Subsets

**Figure 3:**
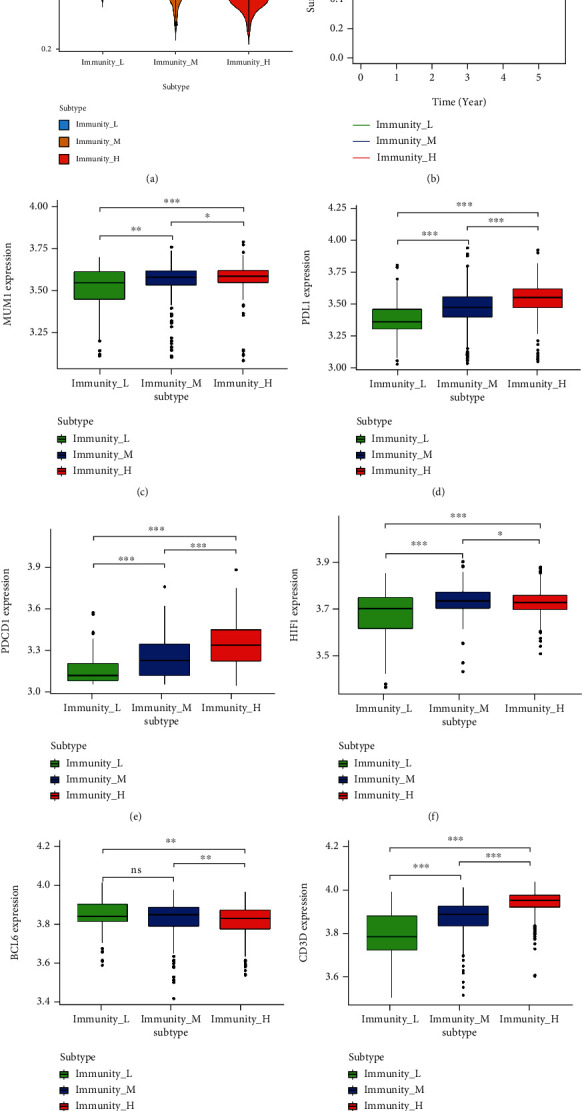
(a) Analysis of differences in tumor purity between three immune subtypes. Tumor purity was importantly more down in Immunity_H group and importantly more excellent in Immunity_L (*p* < 0.001, Kruskal-Wallis test). (b) Survival analysis of three immune subgroups. The survival curves of Immunity_L, Immunity_M, and Immunity_H subgroups were significantly different (*p* = 4.396E − 08). It also proved that immune grouping had a good predictive effect on the survival of DLBCL. Patients in Immunity_H had the best prognosis, patients in Immunity_L had got the poorest prognosis, and the Immunity_M was between them. (c–h) The expression of PD-1, PD-L1, CD3D, HIF1A, IRF4, and other genes was meaningfully correlated with the immune subgroup. The expressions of PD-1, PD-L1, CD3D, HIF1A, IRF4, and other genes were meaningfully dissimilar between Immunity_L and Immunity_H (ANOVA text, *p* < 0.001).

Next, three immune subtypes' prognostic value was measured by us on patient survival. It was discovered that the survival curves of the three subgroups Immunity_H, Immunity_M, and Immunity_L were statistically significantly different (*p* = 4.396e − 08). It also demonstrated that immunophenotyping was a good predictor of survival in DLBCL. Patients in Immunity_H group had the best prognosis, those in Immunity_L group had the worst prognosis, and those in Immunity_M group were in between, as shown in (Figure 3(b)).

### 3.3. Exploration of Immune Subtype-Related Markers

In addition, we also explored the connection during the expression of PD-1, PD-L1, CD3D, HIF1A, and IRF4 genes and immune subgroups. These results showed that the expression of PD-1, PD-L1, CD3D, HIF1A, IRF4, and other genes were meaningfully different in both Immunity_H and Immunity_L groups (ANOVA text, *p* < 0.001), as shown in (Figures 3(c)–3(h)). The results of this study strongly support that the immune microenvironment affects action of immune checkpoint inhibitors in cancer patients, and it also sounds an alarm for the development of new immune checkpoint inhibitors, which cannot ignore the important role of immune microenvironment in novel immunotherapy.

### 3.4. HLA Genes Were Meaningfully Correlated with Immune Subsets

**Figure 4:**
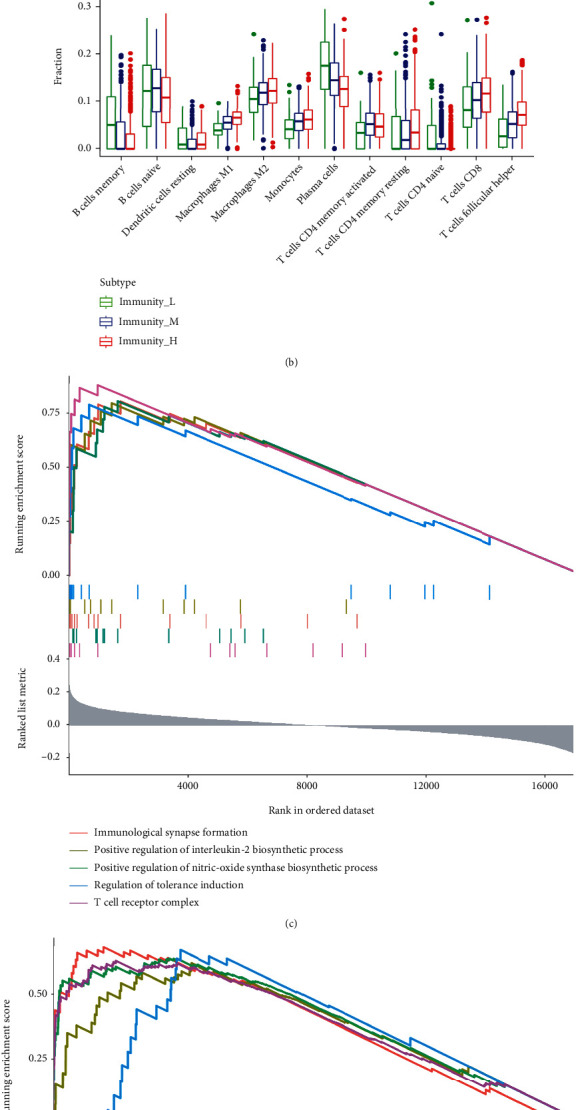
(a) Immune subsets were significantly associated with HLA family genes. Among 24 HLA-related genes, only five genes, HLA-G, HLA-DRB6, HLA-DPB2, HLA-DOB, and HLA-B, were not significant in the distribution of immune subsets. The remaining HLA family members were statistically distributed in the immune subgroups (*p* < 0.06). (b) Immune subtypes were significantly associated with immune cell infiltration. Monocytes, M1 macrophages, M2 macrophages, CD8^+^ T cells, CD4^+^ memory-activated T cells, and follicular helper T cells were substantially high up in the Immunity_H group than in the Immunity_M groups and Immunity_L. The results of B cells naive, B cells memory, plasma cells, and CD4^+^ naive T cells in Immunity_L were considerably more excellent than those in Immunity_M and Immunity_H. (c and d) GO and KEGG analysis differential gene enrichment analysis of Immunity_H and Immunity_L groups.

To test immune-related genes' expression in each subgroup, HLA genes' expression is then explored by us in three immune subgroups. “∗∗∗,” “∗∗,” “∗,” and “ns”, respectively, based on one-way ANOVA (*p* < 0.001, *p* < 0.01, *p* < 0.05, and *p* < 1). These consequences demonstrated that HLA family genes' expression in the Immunity_H was importantly more excellent than that in Immunity_M and Immunity_L, and it was the most down in Immunity_L (Figure 4(a)). Among 24 HLA-related genes, only HLA-G, HLA-DRB6, HLA-DPB2, HLA-DOB, and HLA-B genes had no significance in immune subgroup distribution. The distribution of other HLA family members in immune subgroup was statistically significant (*p* < 0.05).

### 3.5. Immune Subtypes Were Correlated with Immune Cell Infiltration Importantly

To further investigate the important function of tumor microenvironment in DLBCL, the ratio of 22 human immune cell subsets in DLBCL was assessed using the CIBERSORT package in R software. The results revealed that monocytes, M1 macrophages, M2 macrophages, CD8^+^ T cells, CD4^+^ memory-activated T cells, and follicular helper T cells were importantly high up in Immunity_H than Immunity_L, and the consequences of B cells naive, B cells memory, plasma cells, and CD4^+^ naive T cells in Immunity_H groups and Immunity_M were importantly more down than the Immunity_L group (Figure 4(b)).

### 3.6. KEGG Enrichment Analysis and GO

Based on the improvement scores in each sample, the differential genes in the Immunity_L and Immunity_H groups were screened. (Figure 4(c)) shows correlation to the best 5 pathways with the most excellent GO and (Figure 4(d)) reveals the highest 5 pathways with the most excellent KEGG correlation. KEGG analysis showed that the differential genes in Immunity_H and Immunity_L groups were mainly enriched in allograft rejection, Ferroptosis, PD-1 expression, protein export, and PD-1 checkpoint pathway in cancer. GO analysis showed that the low group differential genes and high group were improved with immunological synapse formation, positive regulation of interleukin-2 biosynthetic process, positive regulation of nitric oxide synthase biosynthetic process, regulation of tolerance induction, and T cell receptor complex.

### 3.7. PD-L1 Regulates IRF4 Expression in DLBCL

**Figure 5:**
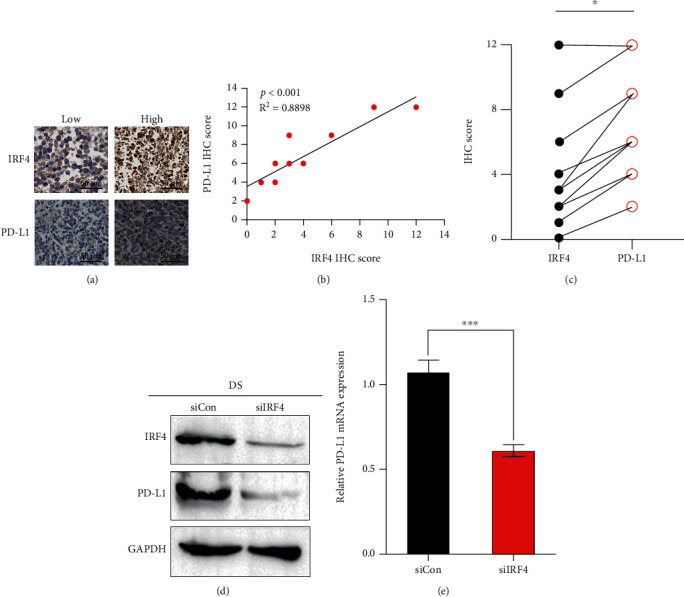
(a) IHC was used to detect the expression of IRF4 and PD-L1 in DLBCL. Two cases were stained with IRF4 and PD-L1 immunohistochemistry. Examine the section under a microscope. (b) The images described are representative of 30 cases of DLBCL. Correlation between IRF4 IHC score and IRF4 IHC score in 30 DLBCL patients, calculated by Spearman's rank correlation methods, in 30 DLBCL cases. (c) We transfected siControl and siIRF4 into DS cell line by transient transfection method. Proteins were collected and lysed, and the displayed proteins were analyzed by western blotting. (d) The expression of PD-L1 was detected by real-time fluorescence quantitative PCR. The error bar represents three separate experiments.

We then valued the expression of PD-L1 proteins and IRF4 by using immunohistochemistry (IHC) in 30 patients diagnosed with DLBCL (Table 2). IRF4 expressions and PD-L1 were notably discovered in the majority of examples in this cohort, whereas PD-L1 overexpression was substantially more usual in cases with excellent IRF4 (Figure 5(a)). PD-L1 IHC score had a good correlation with IRF4 score (*p* < 0.001, Figure 5(b)). Finally, immunoblotting (Figure 5(c)) and real-time quantitative PCR (Figure 4(d)) detection confirmed that knockdown of IRF4 expression in DLBCL could effectively inhibit PD-L1 expression.

### 3.8. Effect of IRF4 on Immune Function

**Figure 6:**
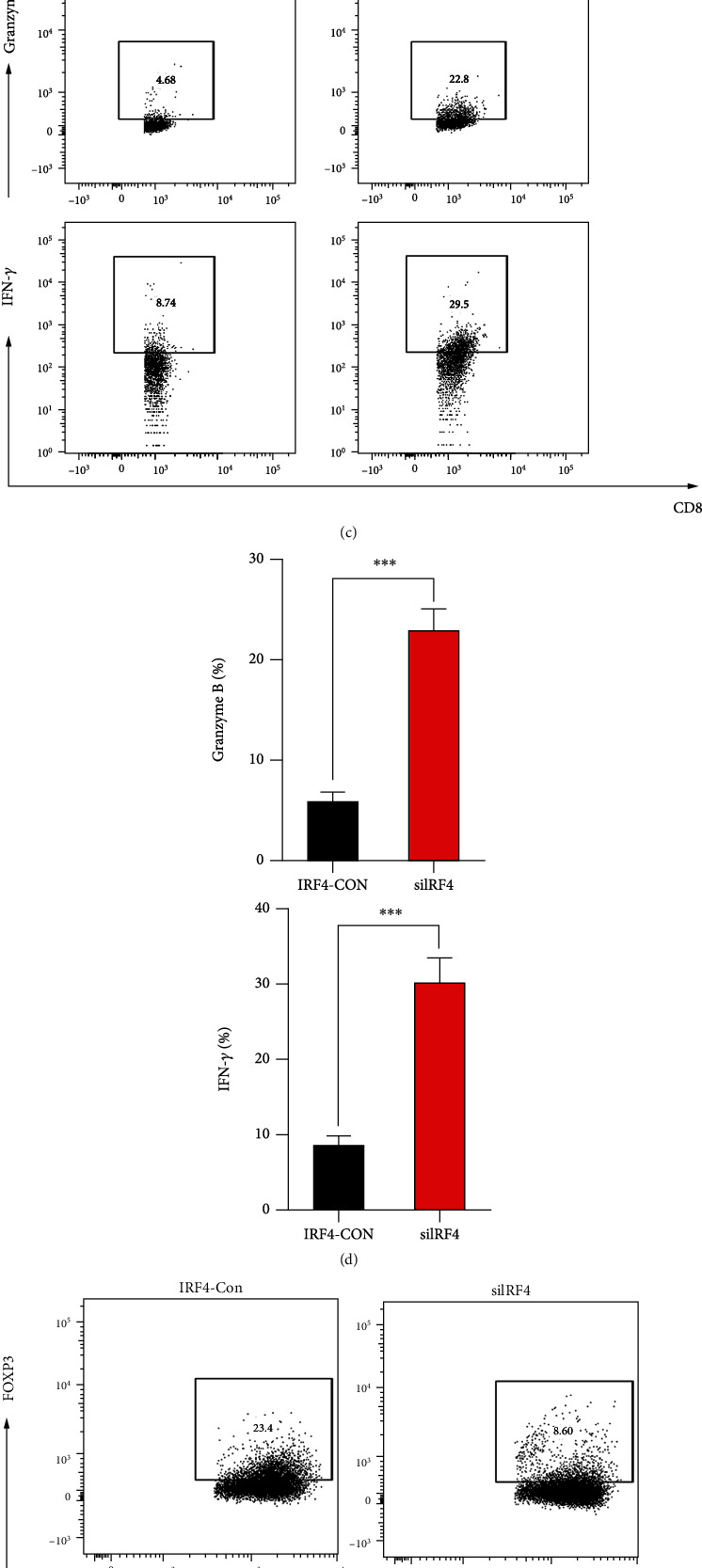
(a and b) PD-L1's flow cytometry analysis in siIRF4 DS cells in relation to restriction with or without IFN-*γ* therapy. (c and d) Flow cytometry was used to analyze Granzyme B^+^ CD8^+^ T cell or IFN-*γ*^+^ CD8^+^ T cell frequencies. (e and f) CD4^+^T cell frequency or Treg (FOXP3) in the PBMC.

In this study, we observed that knocking down IRF4 resulted in reduced PD-L1 induction, and IFN-*γ* induction further confirmed the correlation between IRF4 and PD-L1 (Figures 6(a) and 6(b)). Compared with the control group, DS cells with knockdown IRF4 were coincubated with PBMC, and the immune function of CD8^+^ T cells was detected by using flow cytometry. It was observed that the production of IFN-*γ* and Granzyme B-related molecules of CD8^+^ T cells was more excellent than that of the control group (Figure 6(c)). At the same time, we found that compared to the control group. Knocking down IRF4 can inhibit the differentiation of CD4^+^ T cells into Treg (Figure 6(d)).

## 4. Discussion

Despite the wide variety of clinical, morphological, and molecular parameters used to classify DLBCL today, the 40% survival rate remains poor [17, 18]. At present, genome map has been used to identify and diagnose various molecular subtypes of cancer, and a large amount of evidence indicates that tumor microenvironment plays an important role in tumor genesis, development and treatment [19, 20]. In the meanwhile, immune cells and stromal cells in tumor microenvironment also play a significant part in prognosis at the same time and tumor progression [21, 22]. Therefore, immune-related hierarchical clustering is used to better assess patient outcomes and select therapies that are effective only for specific subtypes of DLBCL.

In our study, we calculated 928 DLBCL samples using ssGSEA and analyzed the enrichment levels of 29 immune-related genomes in each sample. Next, we used unsupervised clustering, which could be clearly based on the three DLBCL subtypes identified by the ssGSEA score: Immunity_High subtype, Immunity_Medium subtype, and Immunity_Low subtype. We used estimation algorithms to calculate each patient's score of immune, stromal, and tumor purity. Analysis showed that of the three subtypes, Immunity_High was connected with importantly more excellent prognosis and accommodated more stromal cells and immune cells than the other groups, showing increased activity in this subgroup. In addition, we discovered the expression of PD-1, PD-L1, CD3D, HIF1A, and IRF4 genes were substantially different in both Immunity_L groups and Immunity_H (ANOVA text, *p* < 0.001).

Class L human leukocyte antigen is an intracellular peptide that can be recognized by T cells on the cell surface. Changes in the HLA gene may alter the ability to express neoantigens and thus affect immune escape. Numerous studies have shown that HLA alterations are strongly associated with cancer prognosis and treatment. In our research, HLA family genes' expression was importantly higher in Immunity_H than in Immunity_L and Immunity_M.

At the same time, an increasing number of researches have illustrated a correlation between the treatment responsiveness and prognosis of tumor patients and the level of immune cell infiltration [23]. We used the CIBERSORT package in R software to evaluate 22 human immune cell subpopulations' part in DLBCL. We discovered significant differences in the level of immune cell infiltration and the proportion of immune infiltrating cell types by immune subtype grouping through our analysis [24]. For instance, the highest proportions of CD8^+^ T cells and CD4^+^ memory T cells were discovered in Immunity_H. Meanwhile, immune checkpoints' role is to exert antitumor impacts by increasing the role of CD4^+^ T and CD8^+^ T cells [25, 26]. It has been reported in the past that CD8^+^ T cell infiltration degrees are positively correlated with cancer prognosis after immunotherapy in various kinds of solid tumors. We found by further study that monocytes, M1 macrophages, M2 macrophages, CD8^+^ T cells, CD4^+^ memory-activated T cells, and follicular helper T cells were meaningfully high up in Immunity_H than these consequences of B cells naive, B cells memory, plasma cells, and CD4^+^ naïve which were meaningfully high up in Immunity_L than in Immunity_H and Immunity_M. Besides, the CD8^+^/Treg ratio was considerably high up in Immunity_High than in Immunity_Low. This indicates that Immunity_High has higher immune response and stronger antitumor activity [27–29].

IRF4/MUM1, a member of the IRF family, is specifically expressed in lymphocytes and is involved in immune regulation through a series of signal transduction actions. Previous studies have shown that abnormal IRF4 expression can be used as a diagnostic and prognostic marker for various hematologic malignancies. IRF4 was described by Qian et al. as a negative prognostic factor for non-small-cell lung cancer [30]. Our study found that IRF4 (MUM1) was an immunotherapeutic target and a potential prognostic marker for DLBCL. We first demonstrated in DLBCL that IRF4 can upregulate the PD-L1 expression of tumor cells. What is more, on the one hand, the high expression of IRF4 in tumor can inhibit function of effector T cells and on the other hand increases the proportion of immunosuppressive cells Treg, which promote the immune escape of cancer cells. Our research showed that it was possible to inhibit the expression of IRF4 in tumor cells and relieve the immunosuppressive effect to achieve the effect of treating DLBCL.

In our study, we found IRF4's expression was meaningfully high up in Immunity_H than in Immunity_M and Immunity_L; meanwhile, we verified the positive correlation between IRF4 and PD-L1 and demonstrated that IRF4 could enhance immunosuppressive effect of tumor microenvironment. These researches showed that it was possible to inhibit the expression of IRF4 in tumor cells and relieve the immunosuppressive effect to achieve the effect of treating DLBCL.

## Figures and Tables

**Table 1:** Dataset related information.

Characteristics		GSE117556 (*n* = 928) (%)
Age	<=60	332 (35.8)
	>60	596 (64.2)
Subtype	ABC	274 (26.3)
	GCB	475 (51.2)
Stage	Stage I-II	268 (30.8)
	Stage III-IV	638 (68.8)
ECOG	<=1	823 (88.7)
	>1	105 (11.3)
LDH	<500	589 (63.4)
	> =500	339 (36.5)
IPI	<=2	482 (51.9)
	>2	446 (48.1)

**Table 2:** Detailed information of tissue array used in IHC.

Age(*n* = 30)	Gender	TNM	IHC store (IRF4)	IHC store (PD-L1)
72	Female	T3N1M0	6	9
74	Female	—	9	12
50	Male	—	1	4
60	Male	—	4	6
38	Male	—	0	2
64	Female	T2N0M0	2	6
73	Male	T2N0M0	6	9
55	Male	T2N3M0	3	9
53	Male	T2N2M0	2	6
73	Female	T1N0M0	4	6
50	Female	T1N1M0	1	4
81	Female	T1EN2M0	6	9
72	Male	T1EN1M0	9	12
55	Male	T2N1M0	9	12
58	Male	T1N0M0	2	4
81	Female	T1N0M0	2	4
57	Male	T1N0M0	1	4
61	Male	T1N0M0	4	6
52	Female	T2N1M0	12	12
65	Male	T3N3M0	12	12
78	Male	T3N1M0	3	6
32	Female	T1N0M0	2	6
60	Male	T2N0M0	4	6
73	Male	T3N1M0	12	12
47	Male	—	12	12
65	Female	—	2	4
64	Male	T2N1M0	12	12
75	Male	T1N0M0	9	12
58	Male	—	9	12
56	Male	T3N0M0	9	12

## Data Availability

The data used to support the findings of this study are available from the corresponding author upon request.

## Supplementary Materials

Supplementary Materials The supplementary table shows 29 immune-related gene sets.
