# Supplementary material for: Immunogenomic-Based Analysis of Hierarchical Clustering of Diffuse Large Cell Lymphoma
Source: J Immunol Res. 2022 Aug 9;2022:9544827. doi: 10.1155/2022/9544827 (PMC9381292; doi:10.1155/2022/9544827)
Supplement: Supplementary Materials — The supplementary table shows 29 immune-related gene sets. [file 9544827.f1.pdf]

aDCs	CD83	LAMP3	CCL1					
APC_co_in	C10orf54	CD274	LGALS9	PDCD1LG2	PVRL3			
APC_co_sti	CD40	CD58	CD70	ICOSLG	SLAMF1	TNFSF14	TNFSF15	TNFSF18
B_cells	BACH2	BANK1	BLK	BTLA	CD79A	CD79B	FCRL1	FCRL3
CCR	CCL16	TPO	TGFBR2	CXCL2	CCL14	TGFBR3	IL11RA	CCL11
CD8+_T_ce	CD8A							
Check-poi	IDO1	LAG3	CTLA4	TNFRSF9	ICOS	CD80	PDCD1LG2	TIGIT
Cytolytic_a	PRF1	GZMA						
DCs	CCL17	CCL22	CD209	CCL13				
HLA	HLA-E	HLA-DPB2	HLA-C	HLA-J	HLA-DQB1	HLA-DQB2	HLA-DQA1	HLA-DQA1
iDCs	CD1A	CD1E						
Inflammati	CCL5	CD19	CD8B	CXCL10	CXCL13	CXCL9	GNLY	GZMB
Macropha	C11orf45	CD68	CLEC5A	CYBB	FUCA1	GPNMB	HS3ST2	LGMN
Mast_cells	CMA1	MS4A2	TPSAB1					
MHC_class	B2M	HLA-A	TAP1					
Neutrophil	EVI2B	HSD17B11	KDM6B	MEGF9	MNDA	NLRP12	PADI4	SELL
NK_cells	KLRC1	KLRF1						
Parainflam	CXCL10	PLAT	CCND1	LGMN	PLAUR	AIM2	MMP7	ICAM1
pDCs	CLEC4C	CXCR3	GZMB	IL3RA	IRF7	IRF8	LILRA4	PHEX
T_cell_co-i	BTLA	C10orf54	CD160	CD244	CD274	CTLA4	HAVCR2	LAG3
T_cell_co-s	CD2	CD226	CD27	CD28	CD40LG	ICOS	SLAMF1	TNFRSF18
T_helper_c	CD4							
Tfh	PDCD1	CXCL13	CXCR5					
Th1_cells	IFNG	TBX21	CTLA4	STAT4	CD38	IL12RB2	LTA	CSF2
Th2_cells	PMCH	LAIR2	SMAD2	CXCR6	GATA3	IL26		
TIL	ITM2C	CD38	THEMIS2	GLYR1	ICOS	F5	TIGIT	KLRD1
Treg	IL12RB2	TMPRSS6	CTSC	LAPTM4B	TFRC	RNF145	NETO2	ADAT2
Type_I_IFN	DDX4	IFIT1	IFIT2	IFIT3	IRF7	ISG20	MX1	MX2
Type_II_IFN	GPR146	SELP	AHR					

TNFSF4	TNFSF8	TNFSF9						
HVCN1	RALGPS2							
IL4I1	IL33	CXCL12	CXCL10	BMPER	BMP8A	CXCL11	IL21R	IL17B
CD70	TNFSF9	ICOSLG	KIR3DL1	CD86	PDCD1	LAIR1	TNFRSF8	TNFSF15
HLA-A	HLA-DMA	HLA-DOB	HLA-DRB1	HLA-H	HLA-B	HLA-DRB5	HLA-DOA	HLA-DPB1
IFNG	IL12A	IL12B	IRF1	PRF1	STAT1	TBX21		
MMP9	TM4SF19							
TRANK1	VNN3							
MX2	CXCL9	ANXA1	TLR2	PLA2G2D	ITGA2	MX1	HMOX1	CD276
PLD4	PTCRA							
LAIR1	TIGIT							
TNFRSF25	TNFRSF4	TNFRSF8	TNFRSF9	TNFSF14				
IRF4	PRKCQ	FCRL5	SIRPG	LPXN	IL2RG	CCL5	LCK	TRAF3IP3
CHST2	CTLA4	NFE2L3	LIMA1	IL1R2	ICOS	HSDL2	HTATIP2	FKBP1A
RSAD2	TNFSF10							

TNFRSF9	ILF2	CX3CR1	CCR8	TNFSF12	CSF3	TNFSF4	BMP3	CX3CL1
TNFRSF14	IDO2	CD276	CD40	TNFRSF4	TNFSF14	HHLA2	CD244	CD274

HLA-DRA	HLA-DRB6	HLA-L	HLA-F	HLA-G	HLA-DMB	HLA-DPA1

TIRAP	IL33	PTGES	TNFRSF12A	SCARB1	CD14	BLNK	IFIT3	RETNLB

CD86	MAL	LILRB1	DOK2	CD6	PAG1	LAX1	PLEK	PIK3CD
TIGIT	CCR8	LTA	SLC35F2	IL21R	AHCYL1	SOCS2	ETV7	BCL2L1

BMP5	CXCR2	TNFRSF10[illegible]	BMP2	CXCL14	CCL28	CXCL3	BMP6	CCL21
HAVCR2	CD27	BTLA	LGALS9	TMIGD2	CD28	CD48	TNFRSF25	CD40LG
IFIT2	ISG15	OAS2	REL	OAS3	CD44	PPARG	BST2	OAS1
SLAMF1	XCL1	GPR171	XCL2	TBX21	CD2	CD53	KLHL6	SLAMF6
RRAGB	ACSL4	CHRNA6	BATF	LAX1	ADPRH	TNFRSF4	ANKRD10	CD274

CXCL9	CCL23	IL6	TNFRSF18	IL17RD	IL17D	IL27	CCL7	IL1R1
ADORA2A	VTCN1	CD160	CD44	TNFSF18	TNFRSF18	BTNL2	C10orf54	CD200R1

NOX1	PLA2G2A	IFIT1	IFITM3	IL1RN

CD40	SIT1	TNFRSF4	CD79A	CD247	LCP2	CD3D	CD27	SH2D1A
CASP1	LY75	NPTN	SSTR3	GRSF1	CSF2RB	TMEM184(	NDFIP2	ZBTB38

CXCR4	CXCR2P1	TGFB1I1	IFNGR1	IL9R	IL1RAPL1	IL11	CSF1	IL20RA
TNFSF4	CD200	NRP1						

FYB	ARHGAP3(	ACAP1	CST7	CD3G	IL2RB	CD3E	FCRL3	CORO1A
ERI1	TRAF3	NAB1	HS3ST3B1	LAYN	JAK1	VDR	LEPROT	GCNT1

IL25	TNFRSF4	IL18	ILF3	CCL20	TNFRSF12A	IL6ST	CXCL13	IL12B

ITK	TCL1A	CYBB	CSF2RB	IKZF1	NCF4	DOCK2	CCR2	PTPRC
PTPRJ	IKZF2	CSF1	ENTPD1	TNFRSF18	METTL7A	KSR1	SSH1	CADM1

TNFRSF8	IL6R	BMPR2	IFNE	IL1RAPL2	IL3RA	BMP4	CCL24	TNFSF13B

PLAC8	NCKAP1L	IL7R	6-Sep	CD28	STAT4	CD8A	LY9	CD48
IL1R1	ACP5	CHST7	THADA	CD177	NFAT5	ZNF282	MAGEH1	

CCR4	IL2RA	IL32	TNFRSF10[illegible]	IL22RA1	BMPR1A	CXCR5	CXCR3	IFNA8
HCST	PTPRCAP	SASH3	ARHGAP2[illegible]	LAT	TRAT1	IL10RA	PAX5	CCR7

IL17REL	IFNB1	IFNAR1	TNFRSF1B	CCL17	IFNL1	IL16	IL1RL1	ILK
DOCK11	PARVG	SPNS1	CD52	HCLS1	ARHGAP9	GIMAP6	PRKCB	MS4A1

CCL25	ILDR2	CXCR1	IL36RN	IL34	TGFB1	IFNG	IL19	ILKAP

GPR18	TBC1D10C	GVINP1	P2RY8	EVI2B	VAMP5	KLRK1	SELL	MPEG1

BMP2K	CCR10	ILDR1	EPO	CCR7	IL17C	IL23A	CCR5	IL7
MS4A6A	ARHGAP15	MFNG	GZMK	SELPLG	TARP	GIMAP7	FAM65B	INPP5D

EPOR	CCL13	IL2RG	IL31RA	TNFAIP6	IFNL2	BMP1	IL12RB1	TNFAIP8
ITGA4	MZB1	GPSM3	STK10	CLEC2D	IL16	NLRC3	GIMAP5	GIMAP4

IL4R	TNFRSF6B	TNFAIP8L1	TNFRSF10[illegible]	IFNL3	CCL5	CXCL6	CXCL1	CCR3

IFFO1	CFH	PVRIG	CFHR1

TNFSF11	CSF1R	IL21	IL1RAP	IL12RB2	CCL1	IL17RA	CCR1	IL1RN

TNFRSF11B	TNFRSF14	IL13	IL2RB	BMP8B	CCL2	IL24	IL18RAP	TGFBI

TNFSF10	TNFRSF11/	CXCL5	IL5RA	TNFSF9	IL1RL2	TNFRSF13(	IL36G	IL15RA

TNFRSF21	CXCL8	IL22RA2	TNFAIP8L2	IL18R1	IFNLR1	CXCR6	CCL3L3	TNFRSF1A

IL17RE	IFNGR2	IL17RC	TNFAIP8L3	ILVBL	TGFBRAP1	CCL4L1	CSF2RA	CCRN4L

CCL26	TNFAIP1	CCRL2	IFNA10	TNFRSF17	IFNA13	IL20	IL18BP	CCL3L1

TNFSF12-[illegible]	IL5	IL23R	IL26	TNF	TGFA	CSF2	IL1F10	CXCL17

TNFSF13	IFNA4	IL37	IL12A	IL7R	IFNA1	IL1A	IL4	IL2

CCL22	CSF3R	IL10	IFNK	TGFB2	IL1R2	IL1B	IL17F	IL27RA

IL15	TNFSF8	IL36B	XCL1	CXCL16	TNFRSF19	IL3	CCL3	IFNA2

BMPR1B	IFNA21	TNFSF18	CCL8	IL17RB	TNFRSF25	IL22	IL10RB	IFNAR2

CCL18	IFNA16	CSF2RB	IL36A	TNFAIP3	IL13RA2	IL13RA1	CCR9	TNFRSF10/

IFNA7	IFNW1	XCL2	TNFSF14	CCR2	BMP15	BMP10	CCL15-CC	TGFBR1

IFNA5	BMP7	IFNA14	IL20RB	IL10RA	IFNA17	CCR6	TGFB3	CCL15

CCL4	CCL27	TNFRSF13[illegible]	TNFAIP2	IL31	IL17A	TNFSF15	CCL19	IFNA6

IL9
